# Untargeted Metabolomics Reveal Parenteral Nutrition-Associated Alterations in Pediatric Patients with Short Bowel Syndrome

**DOI:** 10.3390/metabo12070600

**Published:** 2022-06-27

**Authors:** Ying Wang, Yang Liu, Bei Gao, Junkai Yan, Wei Cai, Lu Jiang

**Affiliations:** 1 Division of Pediatric Gastroenterology and Nutrition, Xinhua Hospital, School of Medicine, Shanghai Jiaotong University, Shanghai 200092, China; wangying02@xinhuamed.com.cn (Y.W.); yanjunkai@xinhuamed.com.cn (J.Y.); 2 Shanghai Key Laboratory of Pediatric Gastroenterology and Nutrition, Shanghai 200092, China; 3 Department of Pediatric Surgery, Xinhua Hospital, School of Medicine, Shanghai Jiao Tong University, Shanghai 200092, China; tsmcliuyang@163.com; 4 School of Marine Sciences, Nanjing University of Information Science and Technology, Nanjing 210044, China; wintergb@hotmail.com; 5 Shanghai Institute for Pediatric Research, Shanghai 200092, China

**Keywords:** intestinal failure, cholestasis, amino acid, glutamine, biomarker

## Abstract

Short bowel syndrome (SBS) is a major cause of intestinal failure (IF) that may require long-term parenteral nutrition (PN) support. However, long-term PN is accompanied by severe complications such as catheter-related blood stream infection (CRBSI) and intestinal failure-associated liver disease (IFALD), and it is associated with high healthcare costs. In this study, we characterized the plasma metabolomic profile and investigated the role of metabolism in predicting long-term PN in pediatric patients with SBS. Untargeted metabolomics was performed in plasma samples from 20 SBS patients with PN support: 6 patients had IFALD and 14 patients had no liver disease. As controls, 18 subjects without liver or intestinal diseases were included for the analysis. SBS patients had distinct plasma metabolomic signatures compared to controls, and several pathways associated with amino acid metabolism and cell death were significantly changed. The presence of IFALD in SBS was associated with alterations of metabolites mainly classified as “amino acids, peptides, and analogues” and “benzene and derivatives”. Serum direct bilirubin levels were negatively correlated with levels of uridine, skatole, and glabrol. Importantly, SBS patients with long-term PN showed significantly increased levels of glutamine compared to those in the short-term PN group. Finally, using multivariate logistic regression analysis, we developed a prediction model including glutamine and creatinine to identify pediatric SBS patients who need long-term PN support. These findings underscore the potential key role of the metabolome in SBS with IF and suggest that metabolomic profiles could be used in long-term PN assessment.

## 1. Introduction

Intestinal failure (IF) can be caused by conditions such as short bowel syndrome (SBS), intestinal dysmotility, and congenital enterocyte disorders [1]. With impaired intestinal function, patients with IF need intravenous replenishment to maintain health and growth since their own intake does not meet the minimum requirements for nutrition [2]. Parenteral nutrition (PN) is an important way to provide source of nutrition for patients with IF, though long-term use may lead to sever complications such as catheter-related blood stream infection (CRBSI) and hepatobiliary dysfunction, which is defined as intestinal failure-associated liver disease (IFALD) [3]. Therefore, the successful transition from PN to enteral nutrition (EN) is critical for IF patients as intestinal adaption progresses. Previous studies suggested that a remaining bowel length of <100 cm is a predictor of permanent IF in adult SBS adults, and the presence of terminal ileum and colon enhances weaning from PN and survival probabilities [4]. Although different risk factors have been proposed to influence the PN duration in pediatric patients with SBS-IF, there is no established way to identify patients who need long-term PN support.

Metabolomics is a comprehensive biochemical profiling technique that is used to assess systemic metabolism in a biological sample, reflecting the “net effects” of genetic, transcriptomic, proteomic, and environmental interactions [5,6]. The authors of a recent study performed fecal metabolomics in infants receiving PN, in which they identified 12 sphingomyelin lipids as potential biomarkers for the development of cholestasis in combination with birth anthropometry [7]. However, current studies in SBS have mostly focused on characterizing gut microbial or bile acid profiles [8,9], and little is known about the global metabolomic signature. Thus, characterizing metabolomic profiles in SBS patients is imperative for the translational potential of metabolomics to be fully realized.

In this study, we performed untargeted metabolomics of plasma samples from 20 pediatric SBS patients with PN support and 18 non-SBS controls to characterize metabolomic signatures. By integrating metabolomic findings with clinical parameters, we developed a prediction model to identify pediatric SBS patients who need long-term PN with higher accuracy than a model only based on clinical data.

## 2. Results

### 2.1. Cohorts Studied

The clinical characteristics of SBS patients and non-SBS controls are summarized in Table 1. The median age of patients with SBS was found to be 4.01 months (IQR: 2.42–6.93 months), 70% of SBS patients were male, and 65% of SBS patients were preterm. The median length of remaining small intestine was 60 cm (IQR: 47.50–70.00 cm), and 65% of the patients had intact ileocecal valves. The median duration of PN was 191 days (IQR: 121–247 days) for patients with SBS, and 85% patients were treated with antibiotics. SBS patients and non-SBS controls significantly differed by age (*p* < 0.001), preterm status (*p* = 0.002), and antibiotics (*p* < 0.001).

### 2.2. Metabolomic Profile Is Altered in Patients with SBS

**Figure 1:**
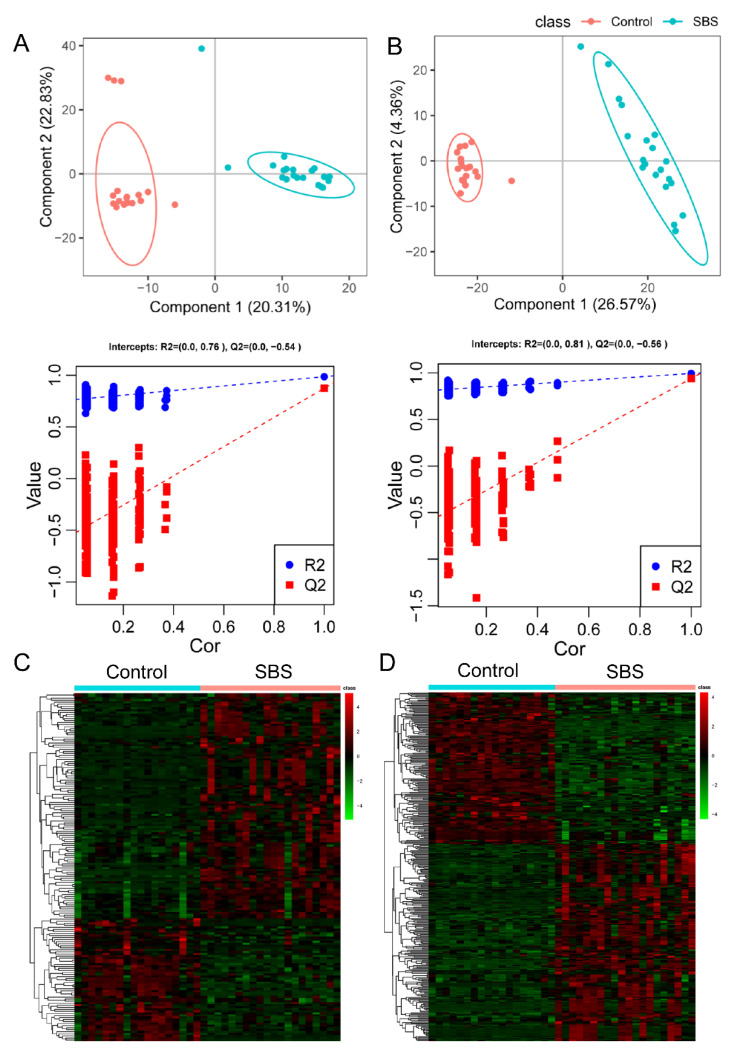
**The metabolomic profile is altered in patients with SBS.** (**A**,**B**) PLS-DA and validation plots from the SBS patients and non-SBS controls in positive mode ((**A**), R2Y = 0.99, Q2 = 0.87) and negative mode ((**B**), R2Y = 0.99, Q2 = 0.94). (**C**,**D**) Hierarchical clustering of differential metabolites in positive mode (**C**) and negative mode (**D**). Data were log2-transformed and zero-mean-normalized, and Euclidean distance was calculated. PLS-DA, partial least squares discriminant analysis.

**Figure 2:**
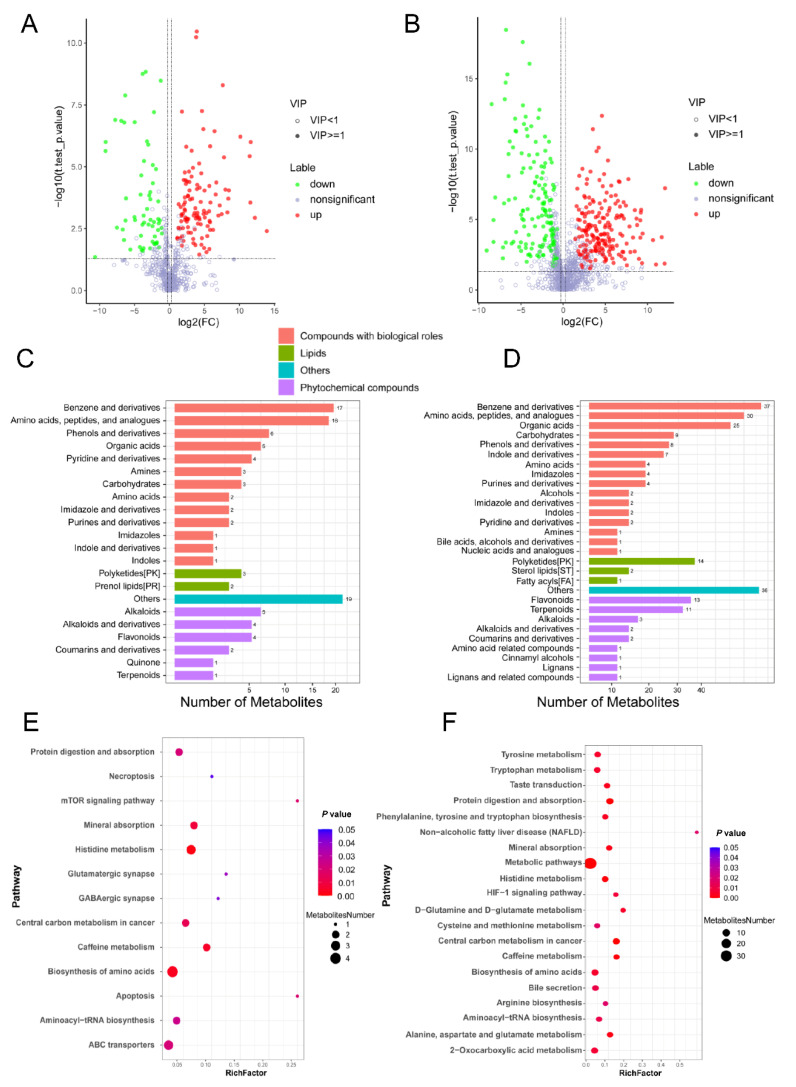
**Functional annotation of differential metabolites identified in the SBS cohort.** (**A**,**B**) Volcano plots showing differential metabolites in patients with SBS compared to non-SBS controls in positive mode (**A**) and negative mode (**B**). Green represents the down-regulated differential metabolites, red represents the up-regulated differential metabolites, and metabolites without statistic difference are labeled gray. (**C**,**D**) Classification of differential metabolites identified in positive mode (**C**) and negative mode (**D**). The X axis represents the number of metabolites in each class, and the Y axis represents the metabolite classification entries. Others means that classification information is the remaining categories. (**E**,**F**) Metabolic pathway enrichment analysis of differential metabolites based on the KEGG database in positive mode (**E**) and negative mode (**F**). RichFactor is the number of differential metabolites annotated to the pathway divided by all identified metabolites annotated to the pathway. The dot size represents the number of differential metabolites annotated to this pathway. Metabolic pathways with *p*-value < 0.05 were significant. VIP, variable importance in the projection.

Overall, 653 and 1131 compounds with identification information were detected by positive and negative modes, respectively. The partial least squares discriminant analysis (PLS-DA) (Figure 1A,B) and hierarchical clustering analysis (Figure 1C,D) of differential metabolites showed that controls and SBS patients differently clustered in both modes. Among all the identified metabolites, 170 (110 up- and 60 down-regulated, positive mode) and 340 (192 up- and 148 down-regulated, negative mode) metabolites were differential metabolites (VIP ≥ 1, FC ≥ 1.2 or ≤0.83, and *p*-value < 0.05; Table S1) when comparing SBS with controls, as shown by volcano plots (Figure 2A,B). The top 10 up- and down-regulated differential metabolites are listed in Table 2, and most of them were drug-derived metabolites as a reflection of antibiotic use in SBS. For compounds with biological roles, differential metabolites were mainly classified as “benzene and derivatives” and “amino acids, peptides, and analogues” in both modes (Figure 2C,D). Lipids were mainly identified by the negative mode, in which polyketides accounted for the most identified secondary metabolites (Figure 2D).

We next performed the pathway enrichment analysis of differential metabolites based on the KEGG database (Figure 2E,F). Overall, pathways associated with amino acid metabolism and cell death were significantly enriched. For example, “histidine metabolism” and “biosynthesis of amino acids” were significantly enriched in both modes. Pathways including “tyrosine metabolism”, “tryptophan metabolism”, “phenylalanine, tyrosine and tryptophan biosynthesis”, “D-glutamine and D-glutamate metabolism”, “cysteine and methionine metabolism”, “arginine biosynthesis”, and “alanine, aspartate, and glutamate metabolism” were exclusively enriched in the negative mode (Figure 2F). In addition, cell death-associated pathways including “necroptosis”, “mTOR signaling pathway”, and “apoptosis” were significantly enriched in SBS compared to controls (Figure 2E).

### 2.3. Development of IFALD in SBS Is Associated with Alterations in the Metabolomic Profiling

**Figure 3:**
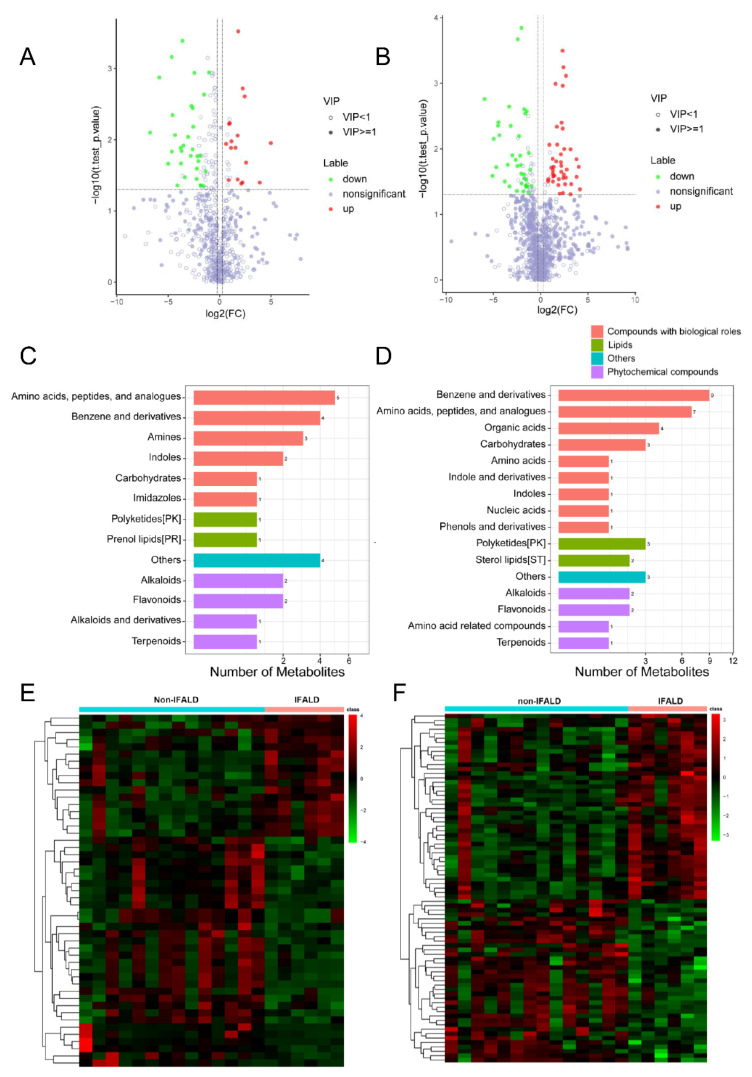
**Altered plasma metabolomic profile in SBS patients with IFALD.** (**A**,**B**) Volcano plots showing differential metabolites in SBS patients with IFALD (n = 6) compared to non-IFALD (n = 14) in positive mode (**A**) and negative mode (**B**). Green represents the down-regulated differential metabolites, red represents the up-regulated differential metabolites, and metabolites without statistic difference are labeled gray. (**C**,**D**) Classification of differential metabolites identified in positive mode (**C**) and negative mode (**D**) SBS patients with IFALD. (**E**,**F**) Hierarchical clustering of differential metabolites in positive mode (**C**) and negative mode (**D**). Data were log2-transformed and zero-mean-normalized, and Euclidean distance was calculated.

Approximately 20–30% of infants and children with SBS who require prolonged PN will develop IFALD [10,11]. In children, IFALD is characterized more by cholestasis. Therefore, we stratified SBS patients into non-IFALD (n = 14) and IFALD (n = 6) groups based on serum direct bilirubin level (≥34.2 μmol/L) to investigate whether the presence of IFALD is associated with alterations in metabolomic profiling. Among all the identified metabolites, 49 (17 up- and 32 down-regulated, positive mode) and 79 (42 up- and 37 down-regulated, negative mode) metabolites were identified as differential metabolites (VIP ≥ 1, FC ≥ 1.2 or ≤0.83, and *p*-value < 0.05; Table S2) when comparing IFALD with non-IFALD, as shown by volcano plots (Figure 3A,B). The top 10 up- and down-regulated differential metabolites are listed in Table 3. Similarly, we detected several drug derivatives, such as probucol, 8-hydroxydemethylclomipramine, and chlorcyclizine. Differential metabolites were mainly classified as “benzene and derivatives” and “amino acids, peptides, and analogues” in both modes (Figure 3C,D). Hierarchical clustering analysis showed that IFALD patients clustered differently compared to non-IFALD patients in the SBS cohort (Figure 3E,F).

**Figure 4:**
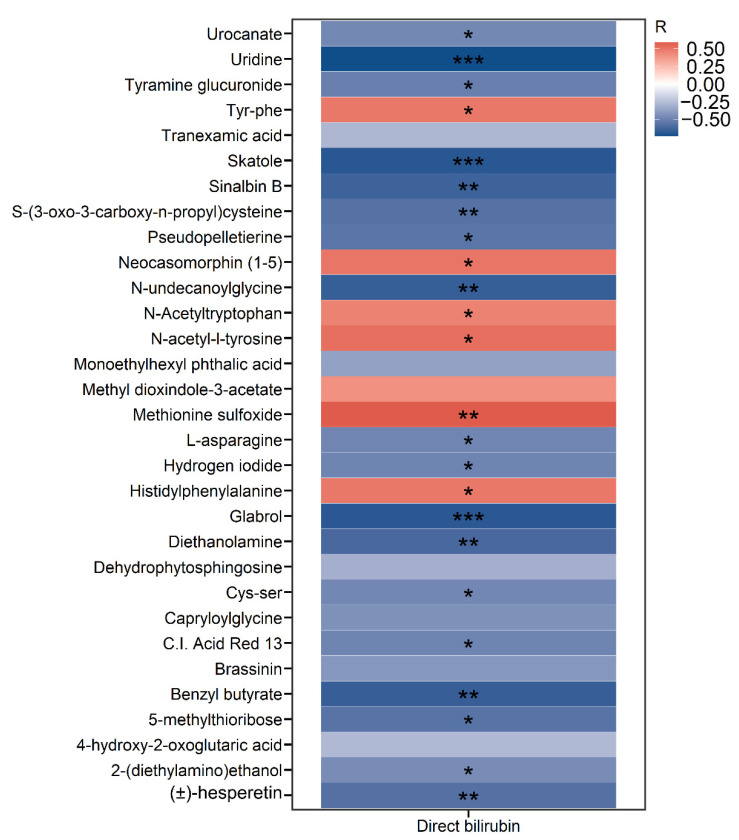
**Serum direct bilirubin levels correlate with differential metabolites in SBS patients.** Heatmap representing color-coded Spearman’s correlations between serum direct bilirubin levels and differential metabolites identified in IFALD (n = 6) patients compared to non-IFALD (n = 14) patients in the SBS cohort. Red color indicates positive correlation, and blue color indicates negative correlation. * *p* < 0.05, ** *p* < 0.01, *** *p* < 0.001.

Next, we investigated the correlations between serum direct bilirubin levels and differential metabolites in SBS patients. Interestingly, the heatmap analysis showed that direct bilirubin was negatively correlated with uridine, skatole, and glabrol but positively correlated with methionine sulfoxide, Tyr-Phe, and histidylphenylalanine, among others (Figure 4).

### 2.4. Metabolites Improve Predictive Accuracy of Long-Term PN in SBS

**Figure 5:**
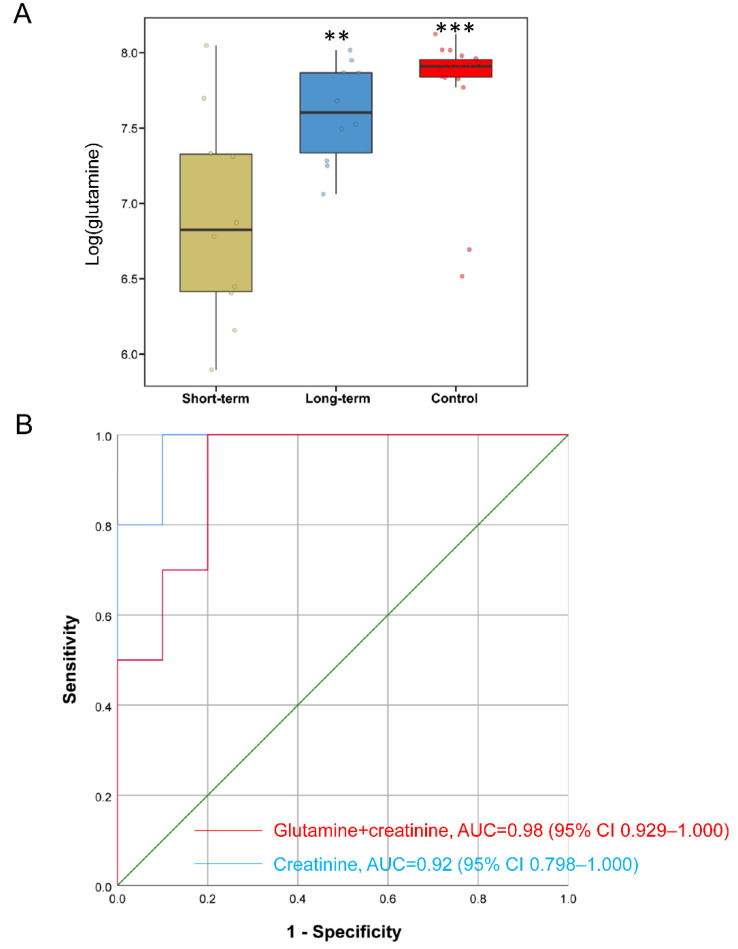
**Prediction of long-term PN using clinical features and glutamine in patients with SBS.** (**A**) Plasma level of glutamine (log transformation) in SBS patients and controls. SBS patients were stratified into two groups (short-term PN and long-term PN) using the median of PN duration as cutoff. (**B**) Receiver operating curves (ROCs) were created based on multivariate regression model to predict long-term PN support in SBS. ** *p* < 0.01, *** *p* < 0.001.

As long-term PN in SBS is an important risk factor for developing sepsis and IFALD [12,13], we aimed to predict whether SBS-IF patients require long-term PN support based on clinical parameters and metabolomic features. We stratified SBS patients into two groups (n = 10 for each group), PN short-term and PN long-term, using the median (191 days) of PN duration as the cutoff. Among all differential metabolites in SBS, univariate regression analyses identified that six of them were significantly associated with long-term PN after adjusting for age, preterm birth status, and antibiotics (Table S3). Interestingly, glutamine level was associated with long-term PN (*p* = 0.05) and was significantly increased in the long-term PN group compared to the short-term PN group (Figure 5A). Therefore, glutamine was included in the prediction model for long-term PN.

For clinical parameters, univariate regression analyses showed that serum creatinine level (*p* = 0.098) was significantly associated with long-term PN (cutoff *p* < 0.1; Supplementary Table S4). Multivariate logistic regression analyses were performed to develop prediction models for long-term PN in SBS. After adjusting for age, preterm birth status, and antibiotics, we found that creatinine alone had an AUC of 0.920 (95% CI: 0.798–1.000), whereas adding glutamine significantly improved the predictive accuracy (likelihood ratio *p*-value = 0.005). This model (glutamine + creatinine) had an AUC of 0.980 (95% CI: 0.929–1.000) for the prediction of long-term PN (Figure 5B). Overall, our data suggest that assessing systemic metabolomics in SBS patients with IF might be helpful to identify patients who need long-term PN support.

## 3. Discussion

In this study, we performed untargeted metabolomics in pediatric SBS patients with IF to demonstrate the translational utility of metabolomics for predicting long-term PN. There were several key findings from this work. First, substantial alterations of metabolites and pathways were associated with amino acid metabolism and cell death in SBS. In SBS patients with IFALD, we observed significant reductions in skatole, glabrol, and uridine that were negatively correlated with serum direct bilirubin. Among all differential metabolites in SBS, glutamine was found to be significantly reduced in SBS patients with the short-term PN group and was negatively correlated with PN duration. Finally, a multivariate logistic regression model using glutamine and creatinine demonstrated a high prediction accuracy for long-term PN in SBS. Since long-term PN is an important risk factor for developing sepsis and IFALD, both of which contribute to the high mortality in patients with SBS-IF, metabolites together with clinical parameters might help to identify patients who need long-term PN support in the future.

In patients with SBS, we observed a substantial proportion of differential metabolites that were classified as amino acids accompanied by alterations of amino acid-associated metabolism pathways. Amino acids are the building blocks of proteins, the disturbance of which may have detrimental effects on life-sustaining chemical processes. In PN-dependent patients, an inadequate supply of amino acids can cause muscle mass reduction and atrophy [14]. In prematurely born infants, PN mixtures enriched in tyrosine, glutamine, taurine, arginine, and cystine are needed to overcome the metabolic immaturity for healthy growth [15]. In addition, we showed the enrichment of glutamine and glutamate-associated metabolism pathways and the significant down-regulation of glutamine levels in SBS. Glutamine is an important amino acid in the modulation of epithelial barrier function under luminal threats [16]. Previous studies showed that glutamine metabolism was impaired in infants underwent small bowel resection [17] and that supplementing dietary glutamine improved gut barrier function in a rat model of SBS [18]. In total parenteral nutrition (TPN) mice, glutamine supplementation prevented the loss of epithelial barrier function and mucosal atrophy accompanied by decreased intestinal permeability and increased expression of tight junction proteins [19]. Furthermore, glutamate is a precursor for the stepwise production of citrulline and proline. Previous studies have described citrulline as a biomarker for remnant enterocyte mass and absorptive function, and its level is reduced in patients with SBS. In patients with SBS-IF, the baseline plasma citrulline level was found to be significantly correlated with small bowel length, and teduglutide treatment for 24 weeks increased the citrulline level [20]. Consistent with previous findings, the perturbation of glutamine metabolism might indicate a promising therapeutic option in pediatric SBS patients.

IFALD has been characterized as a hepatic complication that contributes to significant morbidity in both neonates and adults with IF [21]. The pathogenesis of IFALD has been summarized as the combined effects of the reduced ileal enterocyte production of fibroblast growth factor 19 (FGF19) and the inhibition of hepatic farnesoid X receptor (FXR) that lead to increased bile acid synthesis and retention [22]. In our study, we identified dehydrophytosphingosine as the most down-regulated metabolite in the IFALD group. A recent study by untargeted metabolomics using stool samples from infants receiving PN showed that low birth weight, extreme prematurity, longer duration of PN, and greater number of antibiotic courses were all risk factors for developing PN-associated cholestasis (PNAC). Among all stool biomarkers for the early prediction of PNAC, 12 out of 78 were identified as sphingomyelin lipids that were positively correlated with PNAC [7]. Due to the limited number of patients with IFALD, we did not detect significant changes in the classification of sphingomyelin lipids. Among all differential metabolites in IFALD, we demonstrated that uridine, skatole, and glabrol were significantly reduced and negatively correlated with direct bilirubin, suggesting that their deficiency might contribute to the pathogenesis of disease. Recent studies showed that uridine attenuates carbon tetrachloride-induced liver fibrosis in mice. In vitro, uridine treatment inhibited the expression of alpha-smooth muscle actin (α-SMA) and the migration of hepatic stellate cells (HSCs) [23]. Additionally, uridine administration prevented tamoxifen-induced lipid accumulation in mice, possibly by promoting the biosynthesis of membrane phospholipids [24]. Interestingly, we detected a strong negative correlation between direct bilirubin and skatole levels. Although skatole is mostly reported as a fecal metabolite, our data suggest that it could be used as a plasma biomarker for the development of IFALD in SBS. As a tryptophan metabolite with fecal odor, previous studies regarding skatole were mostly focused on pigs [25]. Skatole has been detected in feces, urine, adipose tissue, and plasma, and its concentration can be affected by feeding strategies and additives [26]. In addition, Deng et al. [27] compared the plasma skatole levels between healthy pregnant women and pregnant women with hepatitis B virus (HBV) infection. They found that plasma skatole levels were significantly different between two groups. In pregnant women with HBV infection, the concentrations of skatole compounds were positively associated with transaminase levels. Future studies are needed to validate the associations between skatole levels and the development of IFALD in a larger cohort and by targeted metabolomics.

Severe SBS is a major cause of IF that may be reversible, depending on anatomy and intestinal adaptation. PN is required for IF patients to provide nutritional support, but the development of severe complications has been a persistent concern [3]. It has been reported that SBS patients at age <1 year have high fluid and nutritional requirements for normal growth and development, and they are more susceptible to PN-associated complications [28]. Therefore, it is imperative to identify SBS-IF patients who need long-term PN. In adults, a previous study by Messing et al. [28] showed that a small bowel length of less than 100 cm is highly predictive of permanent IF. After 2 years of PN, the probability of permanent IF is 94%. In neonates with SBS, survivors with >50 cm of residual bowel had an 88% chance to wean off from PN in 1 year [29]. Importantly, a preserved ileocecal valve in SBS patients was found to be associated with shorter PN duration [30]. Although it is critical for IF patients to wean off PN as intestinal adaptation progresses, this process is highly individualized and complex, and more than 90% of patients who cannot be weaned in 2 years eventually become PN-dependent [4]. In our study, we showed that the level of creatinine was significantly associated with long-term PN. As creatinine is not included in PN solution, the entire creatinine requirement results from de novo synthesis from arginine. In a piglet mode of TPN, creatinine supplementation was found to result in greater protein synthesis in the liver and kidney and lower liver cholesterol, suggesting that the addition of creatinine appears to be necessary in rapidly growing neonates [31]. Consistently, our model suggests that a lower creatinine level might be an indicator of a long-term PN requirement, but more research is needed to investigate the role of creatinine in SBS-IF. Since there is no biomarker available to predict long-term PN in patients with SBS-IF, glutamine together with simple clinical parameters might help in the future.

A strength of our study is the very well-characterized study cohort that included both SBS patients and controls. As the first study to evaluate systemic metabolomic profiles in SBS patients, we have provided a valuable tool to non-invasively predict the long-term requirement of PN. Despite the strengths of the reported findings, several limitations should be noted. First, our study was limited by the small sample size in the SBS cohort. Although we had twenty patients with SBS, only six patients developed IFALD. Second, our study showed that a substantial portion of differential metabolites were drug derivatives, possibly due to the medications used by SBS patients. Last, targeted metabolomics and an independent perspective cohort to validate our findings and predictive model are also needed in the future.

## 4. Materials and Methods

### 4.1. Study Cohorts

Plasma samples were collected from 20 patients with SBS and 18 non-SBS controls from January 2018 to March 2021. SBS was diagnosed via the extensive surgical resection of intestine and required PN administration since the day of admission to the Division of Pediatric Gastroenterology and Nutrition. PN duration was defined from the day receiving PN to the day of weaning off from PN or the death of the patient, as previously described [32]. For SBS patients, exclusion criteria included: (1) patients with obstructive jaundice, suspected or identified biliary tract atresia, viral hepatitis, congenital abnormalities associated with metabolism, major chromosomal diseases, cytomegalovirus infection, and congenital/acquired immune deficiency; (2) patients with primary malignancy at the time of SBS occurrence; (3) patients with functional short bowel (e.g., chronic intestinal pseudo-obstruction and congenital SBS); and (4) PN duration of less than 90 days. A complete medication and medical history were collected at admission. IFALD is defined as serum direct bilirubin ≥2 mg/dL (34.2 μmol/L) in SBS patients who have received PN for at least 90 days and in whom other causes of liver diseases have been excluded [33,34].

For non-SBS controls, subjects with impaired gut function or systemic diseases were excluded. The baseline characteristics of the SBS patients and controls are summarized in Table 1. All clinical sample collection was approved by Ethics Committee of Xinhua hospital affiliated with Shanghai Jiao Tong University School of Medicine (XHEC-C-2021-110-1), with informed consent from the parents or legal guardians.

### 4.2. Untargeted Metabolomics and Data Processing

Metabolite extraction was performed as previously described [35,36]. In short, 100 µL plasma samples were extracted by directly adding 300 µL of precooled methanol and acetonitrile (2:1, *v*/*v*), and an internal standard mix was added as a quality control (QC) of sample preparation. After vortexing and sonicating, samples were incubated at −20 °C for 2 h and centrifuged for 15 min at 20,000 rcf. The supernatant was then transferred for vacuum freeze-drying. The metabolites were resuspended in 100 µL of 70% acetonitrile and centrifuged for 15 min at 20,000 rcf, and the supernatants were transferred to autosampler vials for liquid chromatography–tandem mass spectroscopy (LC–MS/MS) analysis. A QC sample was prepared by pooling the same volume of each sample to evaluate the reproducibility of the whole LC–MS/MS analysis. The metabolite extracts were analyzed on a Waters 2D UPLC (Waters, Milford, MA, USA) coupled to a Q Exactive mass spectrometer (Thermo Fisher Scientific, Waltham, MA, USA) with a heated electrospray ionization (HESI) source. Chromatographic separation was performed on a Waters ACQUITY UPLC BEH Amide column (1.7 μm, 2.1 mm × 100 mm, Waters, USA) with the temperature maintained at 30 °C. Mass spectrometry analysis was conducted in the positive and negative ion modes.

Raw data were imported into Compound Discoverer 3.1 (Thermo Fisher Scientific, USA) for data processing, including peak extraction, retention time correction, additive ion pooling, missing value filling, peak labeling, and metabolite identification. The metabolites were identified through the combined results of the BGI Library (BGI inhouse-developed standard library), mzCloud, and ChemSpider (HMDB, KEGG, LipidMaps) databases. The Kyoto Encyclopedia of Genes and Genomes (KEGG) and the Human Metabolome Database (HMDB) databases were referred to classify and annotate the identified metabolites in order to understand the classification of the metabolites. MetaX [37] was used for statistical analysis, including partial least squares discriminant analysis (PLS-DA) and metabolic pathway analysis. Differential metabolites were selected based on the variable importance in projection (VIP) of the first two principal components of the PLS-DA model ≥1, fold change ≥ 1.2 or ≤0.83, and *p*-value < 0.05.

### 4.3. Statistical Analysis

All statistical analyses were performed using packages in R statistical software (http://www.R-project.org) or SPSS version.26 (IBM corporation, Chicago, IL, USA). Two groups were compared using the two-sided Mann–Whitney U test. Comparisons among the three groups were conducted using one-way analysis of variance (ANOVA) with Tukey’s post hoc test. Spearman’s correlation was performed using the corr.test function in R to calculate the correlations. All statistics were two-sided. As long-term PN is associated with the development of several complications, we stratified SBS patients into two groups (short-term PN and long-term PN) using the median of PN duration as the cutoff. The two missing values of creatinine were filled with the mean value. Univariate regression analyses were performed for differential metabolites and clinical parameters to determine the associations with long-term PN. For differential metabolites, each variable was separately tested after adjusting for age, preterm birth, and antibiotics. Significant metabolomic features (*p*-value < 0.05) and clinical parameters (*p*-value < 0.1) were included in the multivariate logistic regression analysis to develop prediction models for long-term PN. The likelihood ratio test from the “rms” package in R was used to test whether adding metabolomic features to clinical parameters significantly improves the prediction accuracy in multivariate logistic regression models [38]. Receiver operating characteristic (ROC) analysis was performed based on multivariate logistic regression, and the area under the curve (AUC) was reported.

## 5. Conclusions

In our study, we identified unique changes in the metabolomic profile associated with PN duration in patients with SBS. Our study provides valuable information for characterizing the global metabolomic profile in SBS and identifying patients who need long-term PN. We will validate our findings in a larger cohort of SBS by targeted metabolomics in our future studies.

## Figures and Tables

**Table 1:** Demographic and laboratory parameters of the study cohorts.

Characteristics	Data Not Available	Control	SBS	*p*-Value
**Total n**		**18**	**20**	
**Demographics**				
Age (months)		48 (24.00–84.00)	4.01 (2.42–6.93)	**<0.001**
Sex, male, n (%)		11 (61.1%)	14 (70.0%)	0.815
Preterm birth, n (%)		2 (11.11)	13 (65.0)	**0.002**
Antibiotics, n (%)		0 (0.0)	17 (85.0)	**<0.001**
**Laboratory parameters**				
RSL (cm)			60 (47.50–70.00)	
PN duration (days)			191 (121–247)	
Ileocecal valve, n (%)			13 (65.0)	
Bile acid (μmol/L)		4.25 (2.52–5.00)	3.85 (1.48–17.92)	0.965
Creatinine (μmol/L)	2	31.05 (22.70–37.08)	18.60 (16.60–20.82)	**<0.001**
ALT (U/L)		13.25 (10.57–17.60)	104.00 (75.12–248.00)	**<0.001**
AST (U/L)		42.85 (37.95–48.37)	143.00 (95.75–345.00)	**<0.001**
Sodium (mmol/L)	2		138.50 (135.25–139.95)	
Total bilirubin (μmol/L)		4.65 (3.70–6.22)	34.90 (14.70–92.55)	**<0.001**
Direct bilirubin (μmol/L)		0.00 (0.00–0.00)	0.00 (0.00–39.65)	**0.001**
Albumin (g/L)		46.85 (44.23–48.35)	32.70 (31.15–37.23)	**<0.001**
GGT(U/L)		11.00 (10.00–13.75)	114.50 (67.25–188.75)	**<0.001**
White blood cell count, ×10^9^/L			14.45 (11.33–19.12)	
Platelet counts, ×10^9^/L			341.00 (253.75–362.50)	
Prothrombin (s)			15.90 (14.60–16.95)	
INR			1.45 (1.33–1.54)	

Note: Data are presented as median (IQR). Bold font indicates significance (*p*-value < 0.05). Abbreviations: ALT, alanine aminotransferase; AST, aspartate aminotransferase; GGT, gamma-glutamyl transferase; INR, international normalized ratio; PN, parenteral nutrition; RSL, remaining small intestine length.

**Table 2:** Top 10 differential metabolites in SBS patients compared to controls.

Name	VIP	Ratio	*p*-Value	Up/Down
Fluconazole	2.15	15,234.08	0.004	up
Cefmetazole	1.95	4712.83	0.0011	up
1-(4-methoxyphenyl)-3-pentanyl hydrogen sulfate	3.15	4278.50	0	up
Penicilloic acid	1.34	4117.74	0.0134	up
Midazolam	3.58	3119.08	0	up
Omeprazole	2.51	2981.79	0.0003	up
Alpha-hydroxymidazolam	3.46	2787.79	0	up
Cefuroxime	1.67	1597.61	0.0003	up
2-(4-Methyl-5-thiazolyl)ethyl butanoate	3.48	1117.76	0	up
Benzothiazole	2.26	613.59	0	up
Cystathionine	1.46	−1666.67	0.044	down
Piperine	3.20	−555.56	0	down
Guaiacol sulfate	3.23	−357.14	0	down
Dihydronaringenin-o-sulphate	2.36	−312.50	0	down
hesperetin 3′-O-sulfate	1.92	−312.50	0.0004	down
Methyl indole-3-acetate	2.75	−270.27	0	down
2,4,5-Trimethoxybenzaldehyde	1.23	−142.86	0.0057	down
(e)-4-methoxycinnamic acid	1.54	−140.85	0.001	down
3-(7-hydroxy-4-oxo-4h-chromen-2-yl)phenyl hydrogen sulfate	2.25	−129.87	0	down
5-sulfooxymethylfurfural	3.15	−123.46	0	down

Abbreviations: VIP, variable importance in projection.

**Table 3:** Top 10 differential metabolites in SBS patients with IFALD.

Name	VIP	Ratio	*p*-Value	Up/Down
Probucol	2.3583	13.9615	0.0267	up
8-hydroxydemethylclomipramine	2.2311	8.9335	0.0102	up
Chlorcyclizine	2.368	6.3946	0.0008	up
Neocasomorphin (1–5)	1.8981	5.6374	0.0101	up
Bis(5-hydroxynoracronycine)	1.6297	5.4574	0.0247	up
2-acetamido-1,5-anhydro-2-deoxy-3-o-beta-d-galactopyranosyl-d-arabino-hex-1-enitol	2.8951	4.7293	0.0019	up
Ginsenoyne B	1.7813	4.6596	0.004	up
Hydroxychloroquine	1.5343	4.6471	0.0397	up
Tropicamide	1.7151	4.5564	0.0181	up
Histidylphenylalanine	1.6365	4.4079	0.016	up
Dehydrophytosphingosine	1.3912	−32.3625	0.0216	down
C.I. Acid Red 13	2.3328	−31.1526	0.007	down
Norfentanyl	1.8038	−25.0000	0.0146	down
2-ethoxy-4-(4-methyl-1,3-dioxolan-2-yl)phenol	2.4751	−22.0751	0.0039	down
Benzo[a]pyrene-7,8-dihydrodiol-9,10-oxide	1.6514	−20.5761	0.0062	down
(+/−)-hesperetin	1.8447	−17.1527	0.0439	down
Brassinin	1.8249	−13.9082	0.0315	down
Pseudopelletierine	2.3423	−13.7363	0.013	down
Sinalbin B	1.546	−13.5318	0.0215	down
Skatole	1.0416	−11.5340	0.012	down

Abbreviations: VIP, variable importance in projection.

## Data Availability

The data presented in this study are available in the main article and the Supplementary Materials.

## Supplementary Materials

The following supporting information can be downloaded at: https://www.mdpi.com/article/10.3390/metabo12070600/s1: Table S1: Differential metabolites in SBS patients compared to controls, Table S2: Differential metabolites in patients with IFALD compared to non-IFALD in the SBS cohort, Table S3. Plasma metabolites significantly associated with long-term PN according to univariate regression analysis in SBS cohort, Table S4. Associations between clinical parameters and long-term PN according to univariate regression analysis in SBS cohort; Figure S1. Flow diagram of study design.

## Abbreviations

ALT, alanine aminotransferase; ANOVA, analysis of variance; AST, aspartate aminotransferase; AUC, area under the curve; CI, confidence interval; CRBSI, catheter-related blood stream infection; EN, enteral nutrition; GGT, gamma-glutamyl transferase; HMDB, Human Metabolome Database; IF, intestinal failure; IFALD, intestinal failure-associated liver disease; INR, international normalized ratio; KEGG, Kyoto Encyclopedia of Genes and Genomes; PLS-DA, partial least squares discriminant analysis; PN, parenteral nutrition; QC, quality control; ROC, receiver operating curve; RSL, remaining small intestine length; SBS, short bowel syndrome; TPN, total parenteral nutrition; VIP, variable importance in the projection.

## References

[1] Khalaf R.T., Sokol R.J. (2020). New Insights Into Intestinal Failure-Associated Liver Disease in Children. Hepatology.

[2] Pironi L., Arends J., Baxter J., Bozzetti F., Peláez R.B., Cuerda C., Forbes A., Gabe S., Gillanders L., Holst M. (2015). ESPEN endorsed recommendations. Definition and classification of intestinal failure in adults. Clin. Nutr..

[3] Bielawska B., Allard J.P. (2017). Parenteral Nutrition and Intestinal Failure. Nutrients.

[4] Messing B., Crenn P., Beau P., Boutron-Ruault M.C., Rambaud J.C., Matuchansky C. (1999). Long-term survival and parenteral nutrition dependence in adult patients with the short bowel syndrome. Gastroenterology.

[5] Masoodi M., Gastaldelli A., Hyötyläinen T., Arretxe E., Alonso C., Gaggini M., Brosnan J., Anstee Q.M., Millet O., Ortiz P. (2021). Metabolomics and lipidomics in NAFLD: Biomarkers and non-invasive diagnostic tests. Nat. Rev. Gastroenterol. Hepatol..

[6] Kachroo P., Stewart I.D., Kelly R.S., Stav M., Mendez K., Dahlin A., Soeteman D.I., Chu S.H., Huang M., Cote M. (2022). Metabolomic profiling reveals extensive adrenal suppression due to inhaled corticosteroid therapy in asthma. Nat. Med..

[7] Moutinho T.J., Powers D.A., Hanson G.F., Levy S., Baveja R., Hefner I., Mohamed M., Abdelghani A., Baker R.L., Papin J.A. (2022). Fecal sphingolipids predict parenteral nutrition-associated cholestasis in the neonatal intensive care unit. JPEN J. Parenter. Enter. Nutr..

[8] Pereira-Fantini P.M., Byars S.G., Pitt J., Lapthorne S., Fouhy F., Cotter P.D., Bines J.E. (2017). Unravelling the metabolic impact of SBS-associated microbial dysbiosis: Insights from the piglet short bowel syndrome model. Sci. Rep..

[9] Budinska E., Gojda J., Heczkova M., Bratova M., Dankova H., Wohl P., Bastova H., Lanska V., Kostovcik M., Dastych M. (2020). Microbiome and Metabolome Profiles Associated With Different Types of Short Bowel Syndrome: Implications for Treatment. JPEN J. Parenter. Enter. Nutr..

[10] Pichler J., Horn V., Macdonald S., Hill S. (2012). Intestinal failure-associated liver disease in hospitalised children. Arch. Dis. Child..

[11] Abi Nader E., Lambe C., Talbotec C., Pigneur B., Lacaille F., Garnier-Lengliné H., Petit L.M., Poisson C., Rocha A., Corriol O. (2016). Outcome of home parenteral nutrition in 251 children over a 14-y period: Report of a single center. Am. J. Clin. Nutr..

[12] Buchman A.L., Moukarzel A.A., Bhuta S., Belle M., Ament M.E., Eckhert C.D., Hollander D., Gornbein J., Kopple J.D., Vijayaroghavan S.R. (1995). Parenteral nutrition is associated with intestinal morphologic and functional changes in humans. JPEN J. Parenter. Enter. Nutr..

[13] Xiao Y.T., Cao Y., Zhou K.J., Lu L.N., Cai W. (2016). Altered systemic bile acid homeostasis contributes to liver disease in pediatric patients with intestinal failure. Sci. Rep..

[14] Wu G. (2009). Amino acids: Metabolism, functions, and nutrition. Amino Acids.

[15] van Goudoever J.B., Carnielli V., Darmaun D., Sainz de Pipaon M. (2018). ESPGHAN/ESPEN/ESPR/CSPEN guidelines on pediatric parenteral nutrition: Amino acids. Clin. Nutr..

[16] Wang B., Wu G., Zhou Z., Dai Z., Sun Y., Ji Y., Li W., Wang W., Liu C., Han F. (2015). Glutamine and intestinal barrier function. Amino Acids.

[17] Hankard R., Goulet O., Ricour C., Rongier M., Colomb V., Darmaun D. (1994). Glutamine metabolism in children with short-bowel syndrome: A stable isotope study. Pediatr. Res..

[18] Tian J., Hao L., Chandra P., Jones D.P., Willams I.R., Gewirtz A.T., Ziegler T.R. (2009). Dietary glutamine and oral antibiotics each improve indexes of gut barrier function in rat short bowel syndrome. Am. J. Physiol. Gastrointest. Liver Physiol..

[19] Nose K., Yang H., Sun X., Nose S., Koga H., Feng Y., Miyasaka E., Teitelbaum D.H. (2010). Glutamine prevents total parenteral nutrition-associated changes to intraepithelial lymphocyte phenotype and function: A potential mechanism for the preservation of epithelial barrier function. J. Interferon Cytokine Res..

[20] Jeppesen P.B., Gabe S.M., Seidner D.L., Lee H.M., Olivier C. (2020). Citrulline correlations in short bowel syndrome-intestinal failure by patient stratification: Analysis of 24 weeks of teduglutide treatment from a randomized controlled study. Clin. Nutr..

[21] Guglielmi F.W., Regano N., Mazzuoli S., Fregnan S., Leogrande G., Guglielmi A., Merli M., Pironi L., Penco J.M., Francavilla A. (2008). Cholestasis induced by total parenteral nutrition. Clin. Liver Dis..

[22] Mutanen A., Lohi J., Heikkilä P., Jalanko H., Pakarinen M.P. (2015). Loss of ileum decreases serum fibroblast growth factor 19 in relation to liver inflammation and fibrosis in pediatric onset intestinal failure. J. Hepatol..

[23] Zheng W.V., Li Y., Cheng X., Xu Y., Zhou T., Li D., Xiong Y., Wang S., Chen Z. (2022). Uridine alleviates carbon tetrachloride-induced liver fibrosis by regulating the activity of liver-related cells. J. Cell. Mol. Med..

[24] Le T.T., Urasaki Y., Pizzorno G. (2014). Uridine prevents tamoxifen-induced liver lipid droplet accumulation. BMC Pharmacol. Toxicol..

[25] Wesoly R., Weiler U. (2012). Nutritional Influences on Skatole Formation and Skatole Metabolism in the Pig. Animals.

[26] Vhile S.G., Kjos N.P., Sørum H., Overland M. (2012). Feeding Jerusalem artichoke reduced skatole level and changed intestinal microbiota in the gut of entire male pigs. Anim. Int. J. Anim. Biosci..

[27] Deng L., Zhen Q., Gao J., Jin M., Ding M., Xu B. (2017). Simultaneous determination of plasma indole and skatole in pregnant women with hepatitis B virus infection by high performance liquid chromatography. Chin. J. Chromatogr..

[28] Fullerton B.S., Hong C.R., Jaksic T. (2017). Long-term outcomes of pediatric intestinal failure. Semin. Pediatr. Surg..

[29] Fallon E.M., Mitchell P.D., Nehra D., Potemkin A.K., O’Loughlin A.A., Gura K.M., Puder M. (2014). Neonates with short bowel syndrome: An optimistic future for parenteral nutrition independence. JAMA Surg..

[30] Peters F.B., Bone J.N., Van Oerle R., Albersheim S., Casey L., Piper H. (2022). The Importance of the ileocecal valve and colon in achieving intestinal independence in infants with short bowel syndrome. J. Pediatr. Surg..

[31] Dinesh O.C., Bertolo R.F., Brunton J.A. (2018). Creatine supplementation to total parenteral nutrition improves creatine status and supports greater liver and kidney protein synthesis in neonatal piglets. Pediatr. Res..

[32] Wang P., Wang Y., Lu L., Yan W., Tao Y., Zhou K., Jia J., Cai W. (2017). Alterations in intestinal microbiota relate to intestinal failure-associated liver disease and central line infections. J. Pediatr. Surg..

[33] Lal S., Pironi L., Wanten G., Arends J., Bozzetti F., Cuerda C., Joly F., Kelly D., Staun M., Szczepanek K. (2018). Clinical approach to the management of Intestinal Failure Associated Liver Disease (IFALD) in adults: A position paper from the Home Artificial Nutrition and Chronic Intestinal Failure Special Interest Group of ESPEN. Clin. Nutr..

[34] Klein C.J., Revenis M., Kusenda C., Scavo L. (2010). Parenteral nutrition-associated conjugated hyperbilirubinemia in hospitalized infants. J. Am. Diet. Assoc..

[35] Dunn W.B., Broadhurst D., Begley P., Zelena E., Francis-McIntyre S., Anderson N., Brown M., Knowles J.D., Halsall A., Haselden J.N. (2011). Procedures for large-scale metabolic profiling of serum and plasma using gas chromatography and liquid chromatography coupled to mass spectrometry. Nat. Protoc..

[36] Chen C., Hou G., Zeng C., Ren Y., Chen X., Peng C. (2021). Metabolomic profiling reveals amino acid and carnitine alterations as metabolic signatures in psoriasis. Theranostics.

[37] Wen B., Mei Z., Zeng C., Liu S. (2017). metaX: A flexible and comprehensive software for processing metabolomics data. BMC Bioinform..

[38] Núñez E., Steyerberg E.W., Núñez J. (2011). Regression modeling strategies. Rev. Esp. De Cardiol..

