# Supplementary material for: Untargeted Metabolomics Reveal Parenteral Nutrition-Associated Alterations in Pediatric Patients with Short Bowel Syndrome
Source: Metabolites. 2022 Jun 27;12(7):600. doi: 10.3390/metabo12070600 (PMC9319335; doi:10.3390/metabo12070600)
Supplement: Supplementary file 1 [file metabolites-12-00600-s001.zip › Supplemntary file S1.pdf]

Supplementary File S1

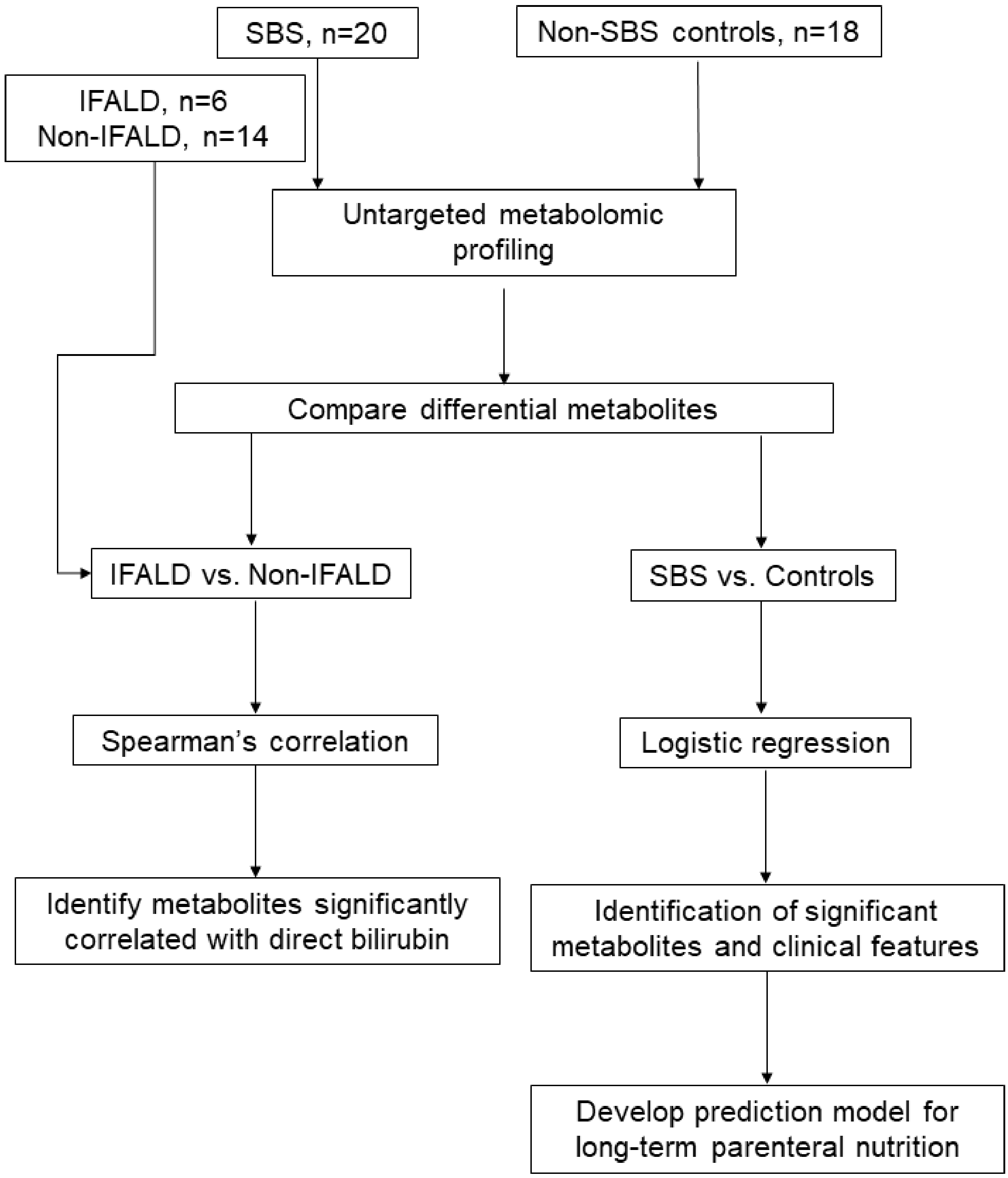

Supplementary Figure S1. Flow diagram of study design. IFALD, intestinal failure-associated liver disease; SBS, short bowel syndrome.

Supplementary Table S3. Plasma metabolites significantly associated with long-term PN according to univariate regression analysis in SBS cohort

Variables	Odds ratio	95%CI	*P* value
Flavidulol C	0.083	0.008-0.829	0.034
Dtdp-4-dehydro-6-deoxy-alpha-d-glucose	21.398	1.140-401.751	0.041
Cyclamic acid	0.000035	1.4601E-9-0.82	0.046
(2z)-2-(4-hydroxybenzylidene) heptyl hydrogen sulfate	9.792	1.029-93.148	0.047
L-pyroglutamic acid	15.678	1.029-238.791	0.048
Glutamine	19.359	0.994-377.037	0.05

The univariate regression analysis models were adjusted for age, preterm, and antibiotics. P value < 0.05 was considered significant. Abbreviations: CI, confidence interval; PN, parenteral nutrition; SBS, short bowel syndrome.

Supplementary Table S4. Associations between clinical parameters and long-term PN according to univariate regression analysis in SBS cohort

Variables	Odds ratio	95%CI	*P* value
Sex	1.000	0.148-6.772	1.000
Age	1.078	0.847-1.373	0.540
Preterm birth	0.643	0.101-4.097	0.640
Antibiotics	2.250	0.170-29.767	0.538
Remaining SI	0.990	0.941-1.041	0.693
Ileocecal valve	1.556	0.244-9.913	0.640
Bile acid	0.972	0.927-1.020	0.249
Creatinine	0.806	0.624-1.041	**0.098**
Sodium	0.990	0.747-1.312	0.943
ALT	0.998	0.990-1.006	0.611
AST	0.996	0.991-1.002	0.245
Total bilirubin	0.991	0.977-1.006	0.245
Direct bilirubin	0.989	0.968-1.011	0.318
Albumin	0.854	0.661-1.103	0.226
GGT	0.993	0.983-1.004	0.217
White blood cell count	0.997	0.900-1.103	0.946
Platelet counts	1.001	0.995-1.007	0.786
Prothrombin	1.057	0.723-1.545	0.773
INR	2.363	0.035-158.019	0.688

Bold font indicates significance (*P* value < 0.05). Abbreviations: ALT, alanine aminotransferase; AST, aspartate aminotransferase; CI, confidence interval; GGT, gamma-glutamyl transferase; HC, healthy control; INR, international normalized ratio; PN, parenteral nutrition; RSL, remaining small intestine length; SBS, short bowel syndrome.
